# What can we do about patients presenting with myeloma and severe renal failure? Observations from the UK MERIT plasma exchange trial

**DOI:** 10.1002/jha2.620

**Published:** 2022-12-07

**Authors:** Judith Behrens, Gill Gaskin, Neil Iggo, Jonathan Barratt, Jane Tighe, Richard Soutar, Gordon Cook, Mary Drake, Curly Morris, Mark Drayson

**Affiliations:** ^1^ Department of Haematology St Helier Hospital Carshalton UK; ^2^ Department of Renal Medicine Hammersmith Hospital London UK; ^3^ Sussex Kidney Unit Brighton and Sussex University Hospitals NHS Trust Brighton UK; ^4^ Department of Cardiovascular Sciences John Walls Renal Unit, Leicester General Hospital Leicester UK; ^5^ Department of Haematology Aberdeen Royal Infirmary Foresterhill UK; ^6^ Department of Haematology Glasgow Royal Infirmary Glasgow Glasgow UK; ^7^ Leeds Cancer Centre St. James University Hospital Leeds UK; ^8^ Haematology Department Belfast City Hospital Belfast UK; ^9^ Centre for Cancer Research and Cell Biology Queens University Belfast UK; ^10^ Clinical Immunology Service University of Birmingham Birmingham UK

**Keywords:** free light chains, myeloma, plasma exchange, renal failure

## Abstract

Myeloma patients presenting with renal failure continue to have a poor prognosis despite significant advances in anti‐myeloma therapy. MERIT was a randomised clinical trial (RCT), set up to evaluate if mechanical reduction of elevated free light chain levels (FLC) would result in clinical benefit. Completion of the planned seven plasma exchanges (PEs) in the first 14 days failed to show, for the exchange group, a greater reduction in FLC or any improvement in dialysis independence at 100 days or subsequently. To improve prognosis for these patients requires earlier diagnosis and prompt anti‐myeloma therapy with effectiveness guided by frequent FLC monitoring.

## 1 SUMMARY

Myeloma patients presenting with renal failure continue to have a poor prognosis similar to those with high‐risk cytogenetics, and their very presentation makes them a difficult group of patients to manage [1, 2]. Myeloma cast nephropathy causes up to 90% of severe acute kidney injury in myeloma patients and is caused by high levels of nephrotoxic monoclonal FLCs [2, 3]. A high proportion of these patients have light chain only (LCO) disease and compared to other paraprotein types LCO patients present at younger age with more renal damage, skeletal fractures and advanced disease stage suggesting delayed diagnosis, and, in older people missed diagnosis [4].

Early diagnosis and reduction of serum FLC levels (sFLC) is associated with renal recovery and improved survival [5, 6]. sFLC can be lowered by stopping secretion with anti‐myeloma therapy and by physical removal, classically by PE [7]. Two RCTs of PE or not in 29 and 21 patients provided conflicting results and a subsequent RCT in 104 patients showed no benefit to renal recovery or patient survival [8, 9, 10]. These trials did not monitor sFLC, and so the efficacy of removal of FLC by PE was unknown, and even combined, the trials are too small to establish the clinical benefit or not of PE.

The UK MERIT trial recruited from February 2004, 78 newly diagnosed myeloma patients with acute renal failure unresponsive to <3 days treatment with fluid and/or treatment of hypercalcaemia with bisphosphonate (creatinine >500 mmol/l, urine output <400 ml/d or requiring dialysis) (Supplementary Material 1). Forty patients were randomised to no PE and 38 to PE; seven treatments by cytocentrifugation or plasma filtration (days 1–14; 4 in days 1–7). All patients received dexamethasone 40 mg days 1–4 and 9–12 and from day 17–100 Vincristine, adriamycin and dexamethasone chemotherapy, or, following protocol amendment in 2005, treatment as specified at randomisation by the treating clinician (Supplementary Material 1).

The primary end point was the proportion of patients alive and dialysis‐independent at 100 days. Secondary endpoints included overall survival, proportion of patients alive and dialysis independent at 6 and 12 months, eGFR at 15 days, 100 days, 6 months and 12 months, change in sFLC between 0 and 15 days, response of myeloma to treatment according to standard criteria at 100 days, 6 and 12 months, renal histology and quality of life. sFLC (mg/l) were measured centrally on days 0, 5, 10, 15 and 100; these levels were compared between the two treatment arms and between patients alive and dialysis‐independent at 100 days and those who were not.

There were 22/38 patients on dialysis in the PE arm with 25/40 in the control arm. The two arms were also well balanced with respect to age, sex, type of myeloma and initial levels of haemoglobin, leucocytes, neutrophils, platelets, albumin, GFR (patients not on dialysis only), beta 2 microglobulin, corrected calcium, sFLC, bone marrow plasma cells and International Staging System (Supplementary Material 2). Sixteen patients had PE by centrifugation and 21 by ultrafiltration (data on one patient missing); 29 patients received the full seven PEs over 14 days and a further eight received 1, 3, 4, 4, 4, 5, 6 and 6 cycles before discontinuation.

**FIGURE 1:**
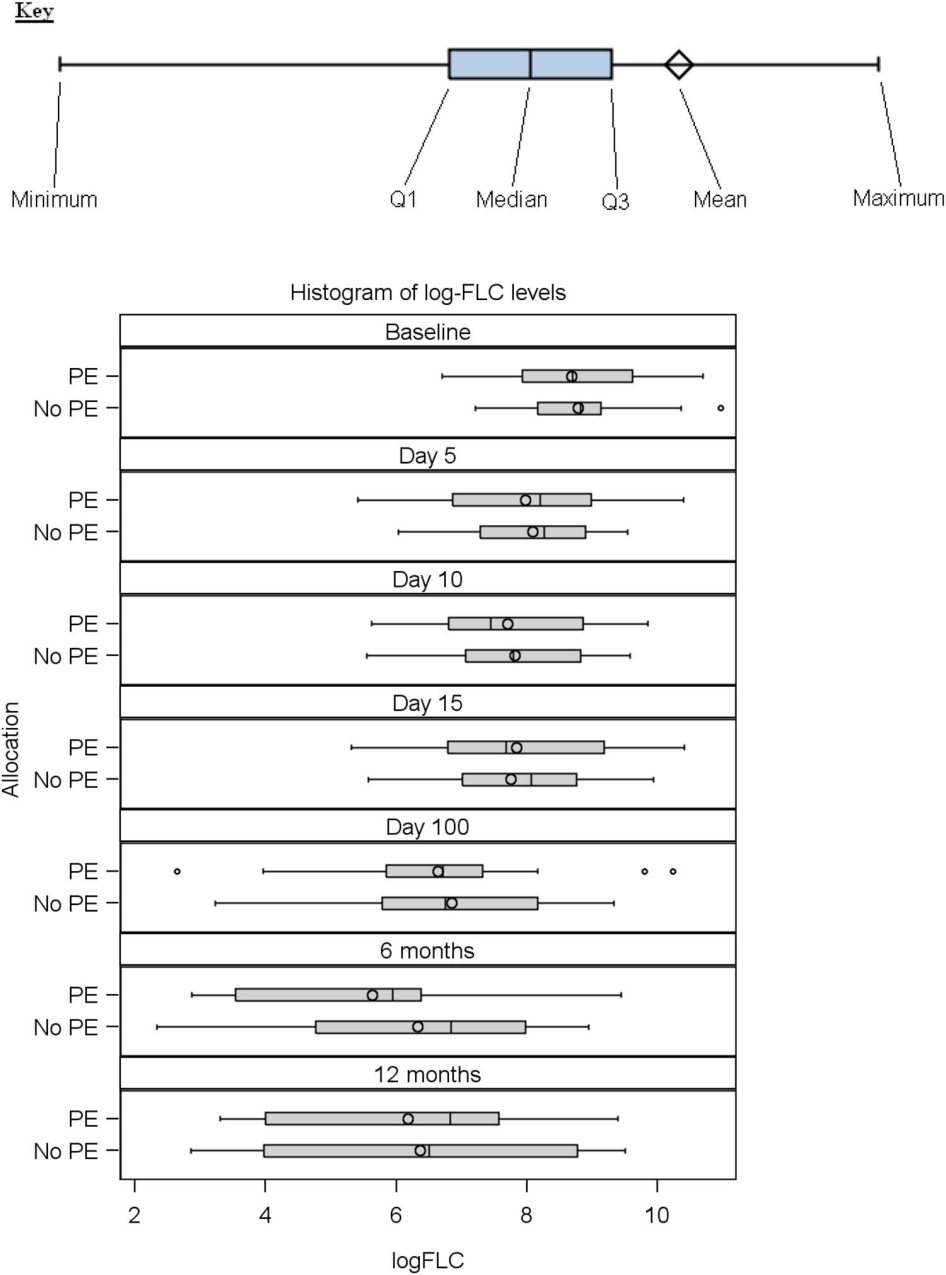
Box plots displaying the log‐malignant serum free light chain results (mg/l) at each time point for plasma exchange or not cohorts (PE No PE)

**FIGURE 2:**
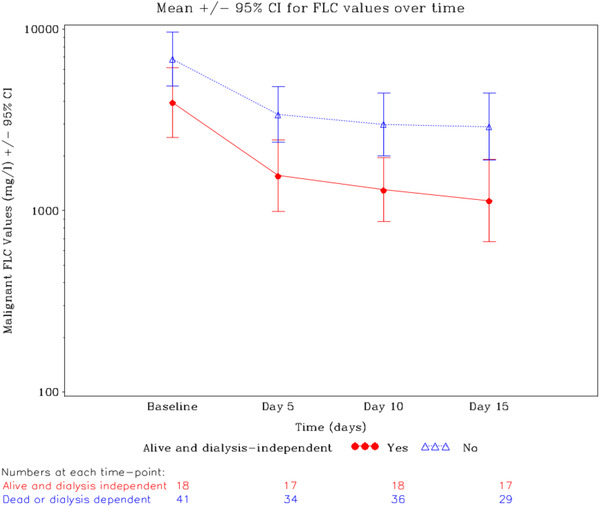
Rapid reduction in the levels of malignant sFLC from trial entry to day 15. The 18 patients who were alive and dialysis independent at 100 days had lower FLC levels *p* = 0.019 than the 34 patients who were dialysis dependent and the seven who died.

With 11 patients alive and dialysis independent in the PE group and 11 in the non‐PE group at day 100, there was no significant benefit for PE (*p* = 0.876), with similar non‐significant differences at 6 and 12 months (Supplementary Material 3). Figure 1 shows malignant sFLC were reduced significantly from base line to day 15 (*p* < 0.0001), with further reduction in time but with no difference between the PE and non‐PE arms (Supplementary Material 3). The greatest drop in sFLC occurred in the first five days (note log scale). For the 18 patients who were alive and dialysis independent at 100 days, malignant sFLC were significantly lower than in the patients who were dialysis dependent or dead (Figure 2, *p* = 0.019). All patients alive and dialysis independent at 100 days showed a response in their sFLC, whereas this was not the case for all dialysis dependent and dead patients (Figure S2B). In a Cox proportional hazards model with Mental Component Score as a continuous variable based on 52 patients who completed baseline QoL assessment before randomization, for every decrease of 1 in the mental component score patients had a decrease of 0.04 in the hazard of death (*p* = 0.037).

## 2 DISCUSSION

While the prognosis for patients presenting with renal failure has improved in line with the general improvement in overall survival for myeloma patients, it is clear there remains an area of largely unmet need for these patients [11]. Renal failure is strongly associated with high sFLC, and sFLC are particularly high in LCO myeloma. In previous MRC UK myeloma RCTs, IgG, IgA and LCO myeloma accounted for 58%, 28% and 14% of 2592 newly diagnosed myeloma patients respectively [4]. Twenty‐eight percent of IgA and IgG myeloma patients did not have significant urinary flc excretion, and within this group only 2% had renal impairment. Eleven percent of IgG and 13% of IgA patients had high levels of urine flc excretion (greater than 12 g/g creatinine), and 48% of these patients had renal impairment, similar to a 54% rate of renal impairment in the 60% of LCO patients with greater than 12 g/l creatinine flc excretion [4]. In a study of 178 patients with biopsy proven cast nephropathy, 48% were LCO patients and as well as FLC levels, extent of cast formation and interstitial fibrosis / tubular atrophy predicted poor renal response [12]. LCO patients account for half of MERIT patients further emphasising the problem of high sFLC and delayed diagnosis [4, 5, 6]. The Birmingham and Oxford groups have shown recently it is possible to speed up the diagnostic process by prompt flagging up of and management of new sFLC results over 500 mg/L [13].

If renal failure cannot be reversed promptly, many patients will remain or become dialysis dependent with the adverse prognosis this implies [5, 6, 7]. This study has shown that physical removal of FLC be PE is by comparison with anti‐myeloma therapy insignificant in lowering sFLC. An alternative to PE is haemodialysis for removal of FLC. High‐flux membranes remove some FLC whilst high cut‐off (HCO; 50 kDa) membranes can provide high clearance rates of FLC. Two RCT of these modalities have shown in 98 and 90 myeloma patients, respectively, with biopsy proven cast nephropathy, no benefit to renal recovery or survival at 3 months although in the first reported study fewer patients in the HCO arm were dialysis dependent at 6 and 12 months [14, 15].

Whilst there is a need for further efforts and studies aimed at this group, there are persisting challenges in recruiting patients to clinical trials in this setting. Such patients are very sick and may struggle to deal with the challenges of participation in a clinical trial at a stage when they have so many other pressing issues to face. Cross‐disciplinary and even cross‐site coordination is required, creating further hurdles to the recruitment of patients and implementation of protocols. MERIT faced the additional issue that after the trial was set up, a range of new therapies were introduced, which were effective in rapidly reducing FLC production, and clinicians understandably felt these new treatments were potentially better for patients, and so recruitment to MERIT slowed. Currently it seems unlikely that further trials involving the laborious and time consuming option of the physical removal of FLC will be undertaken in preference to trials of applying anti‐myeloma therapies that rapidly lower FLC secretion.

It is important therefore to report the trials that have been conducted, despite their limitations, so that clinicians have all the evidence available on which to base decisions on best clinical care of patients. Of the trials investigating physical removal of light chains, MERIT was the first and most detailed in monitoring of the serum flc response, and the demonstration that a positive response is linked to superior outcomes. Close monitoring of such patients during the early days of induction could indicate the need for a change of therapy in refractory patients at an early stage to promote an effective response. Although MERIT did not succeed in recruiting the number of patients that had been intended in the original trial protocol, it has therefore succeeded in adding to the body of evidence about the role of PE in patients with myeloma and severe renal impairment.

With at least six RCTs now showing little evidence of benefit from physical removal of sFLC by PE or HCHD, it seems there is little enthusiasm for this modality in the modern management of myeloma with severe renal failure. The available data indicate that rapid diagnosis and the immediate institution of highly effective anti‐myeloma therapy are the key to improving the outlook for these patients. Monitoring the effectiveness of therapy for lowering sFLC levels in the early days and weeks should guide treatment choices, and this should be a major focus for clinical trials in these patients.

## AUTHOR CONTRIBUTIONS

JB and MD designed the study. CM, JB and MD wrote the manuscript. Dean Langan contributed to statistical analysis and Alexandra Smith performed trial coordination and collected data. The manuscript was approved by all authors

## CONFLICT OF INTEREST

Mark Drayson owns shares in Abingdon Health. All other authors report no conflict of interest.

## ETHICS STATEMENT

The trial was approved by the Eastern Multicentre Research Ethics Committee on 17th July 2003 (REC reference 03/5/023).

## Supporting information

Supporting Information

## Data Availability

Deidentified participant data are available. Any requests for trial data and Supporting Material (data dictionary, Protocol, and statistical analysis plan) will be reviewed by the trial management group in the first instance. Only requests that have a methodologically sound proposal and whose proposed use of the data has been approved by the independent trial steering committee will be considered. Proposals should be directed to the corresponding author in the first instance; to gain access, data requestors will need to sign a data access agreement.

## References

[1] Dimopoulos MA , Terpos E , Chanan‐Khan A , Leung N , Ludwig H , Jagannath S , et al. Renal impairment in patients with multiple myeloma: a consensus statement on behalf of the International Myeloma Working Group. J Clin Oncol. 2010;28:4976–84. 20956629 10.1200/JCO.2010.30.8791

[2] Royal V , Leung N , Troyanov S , Nasr SH , Écotière L , LeBlanc R , et al. Clinicopathologic predictors of renal outcomes in light chain cast nephropathy: a multicenter retrospective study. Blood. 2020;135(21):1833–46. 32160635 10.1182/blood.2019003807 PMC7243151

[3] Hutchison CA , Batuman V , Behrens J , Bridoux F , Sirac C , Dispenzieri A , et al. The pathogenesis and diagnosis of acute kidney injury in multiple myeloma. Nat Rev Nephrol. 2011;8(1):43–51. 22045243 10.1038/nrneph.2011.168 PMC3375610

[4] Drayson M , Begum G , Basu S , Makkuni S , Dunn J , Barth N , et al. Effects of paraprotein heavy and light chain types and free light chain load on survival in myeloma: an analysis of patients receiving conventional‐dose chemotherapy in Medical Research Council UK multiple myeloma trials. Blood. 2006;108(6):2013–9. 16728700 10.1182/blood-2006-03-008953

[5] Hutchison CA , Cockwell P , Stringer S , Bradwell A , Cook M , Gertz MA . et al. Early reduction of serum‐free light chains associates with renal recovery in myeloma kidney. J Am Soc Nephrol. 2011;22:1129–36. 21511832 10.1681/ASN.2010080857 PMC3103732

[6] Hutchison CA , Bladé J , Cockwell P , Cook M , Drayson M , Fermand J , et al. Novel approaches for reducing free light chains in patients with myeloma kidney. Nat Rev Nephrol. 2012;8(4):234–43. 22349488 10.1038/nrneph.2012.14

[7] Dimopoulos MA , Sonneveld P , Leung N , Merlini G , Ludwig H , Kastritis E , et al. International Myeloma Working Group recommendations for the diagnosis and management of myeloma‐related renal impairment. J Clin Oncol. 2016;34(13):1544–57. 26976420 10.1200/JCO.2015.65.0044

[8] Zucchelli P , Pasquali S , Cagnoli L , Ferrari G . Controlled plasma exchange trial in acute renal failure due to multiple myeloma. Kid Int. 1988;33:1175–80. 10.1038/ki.1988.127 3043077

[9] Johnson WJ , Kyle RA , Pineda AA , O'Brien PC , Holley KE . Treatment of renal failure associated with multiple myeloma. Plasmapheresis, hemodialysis, and chemotherapy. Arch Int Med. 1990;150:863–9. 2183734

[10] Clark WF , Stewart AK , Rock GA , Sternbach M , Sutton DM , Barrett BJ , et al. Plasma exchange when myeloma presents as acute renal failure a randomized, controlled trial. Ann Intern Med. 2005;143:777–84. 16330788 10.7326/0003-4819-143-11-200512060-00005

[11] Evison F , Sangha J , Yadav P , Aung YS , Sharif A , Pinney JA , et al. A population‐based study of the impact of dialysis on mortality in multiple myeloma. Br J Haematol. 2018;180(4):588–91. 27766629 10.1111/bjh.14394

[12] Royal V , Leung N , Troyanov S , Nasr SH , Écotière L , LeBlanc R , et al. Clinicopathologic predictors of renal outcomes in light chain cast nephropathy: a multicenter retrospective study. Blood. 2020;135(21):1833–46. 32160635 10.1182/blood.2019003807 PMC7243151

[13] Rana R , Pratt G , Cook M , Drayson MT , Ramasamy K , Sadler R , et al. Improving the diagnostic pathway in patients presenting with acute kidney injury secondary to de novo multiple myeloma: a short report. BMJ Open Quality. 2021;10:001085. 10.1136/bmjoq-2020-001085 PMC8258662 34226245

[14] Bridux F , Carron P , Pegourie B , Alamartine E , Augeul‐Meunier K , Karras A , et al. Effect of high‐cutoff hemodialysis vs conventional hemodialysis on hemodialysis independence among patients with myeloma cast nephropathy: a randomized clinical trial. JAMA. 2017;318:2099–110. 29209721 10.1001/jama.2017.17924 PMC5820717

[15] Hutchison CA , Cockwell P , Moroz V , Bradwell AR , Fifer L , Gillmore JD , et al. High cutoff versus high‐flux haemodialysis for myeloma cast nephropathy in patients receiving bortezomib‐based chemotherapy (EuLITE): a phase 2 randomised controlled trial. Lancet Haematol. 2019;6(4):217–28. 10.1016/S2352-3026(19)30014-6 30872075

