# Supplementary material for: What can we do about patients presenting with myeloma and severe renal failure? Observations from the UK MERIT plasma exchange trial
Source: EJHaem. 2022 Dec 7;4(1):246–50. doi: 10.1002/jha2.620 (PMC9928667; doi:10.1002/jha2.620)
Supplement: Supplementary file 1 — Supporting Information [file JHA2-4-246-s001.docx]

**Figure 1** Box plots displaying the log-malignant serum free light chain results (mg/l) at each time point for Plasma exchange or not cohorts (PE No PE).


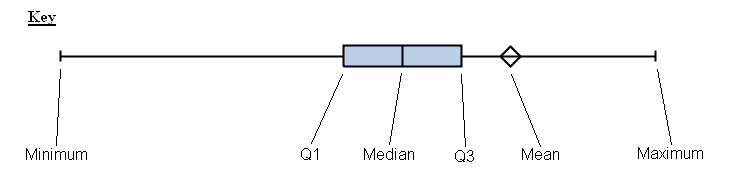

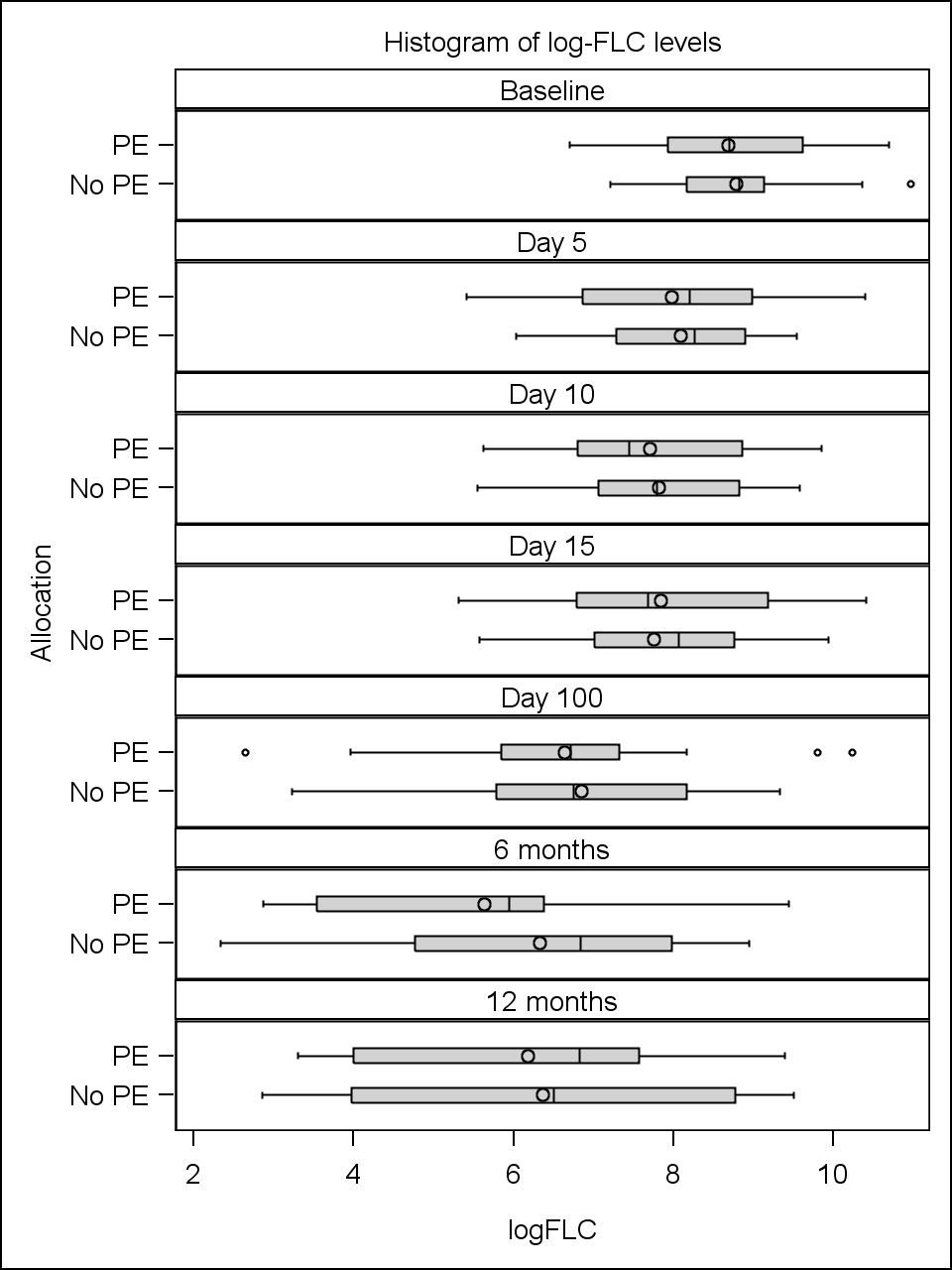


**Figure 2 Rapid reduction in levels of malignant sFLC from trial entry to day 15**

##
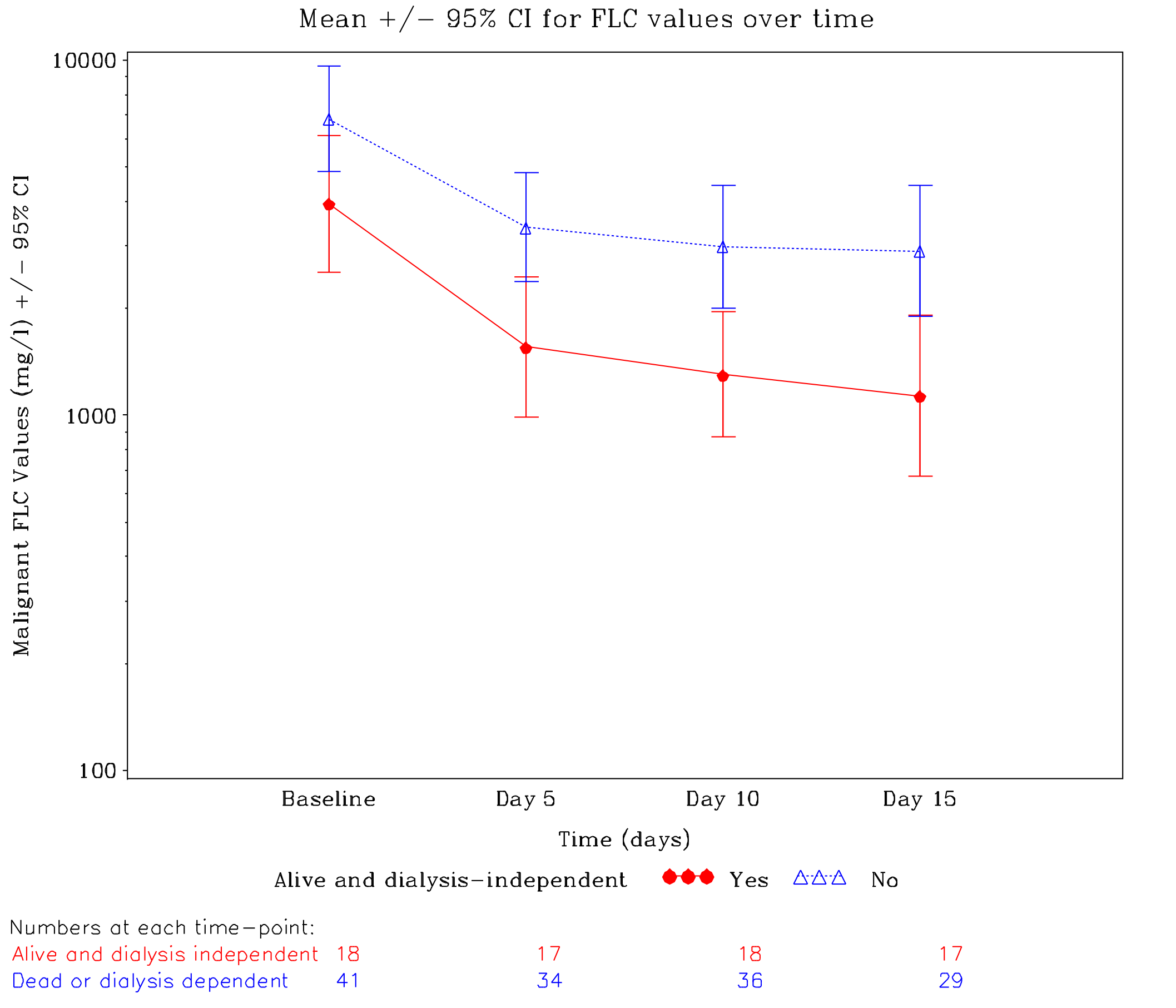


## The 18 patients who were alive and dialysis independent at 100 days had lower flc levels p=0.019 than the 34 patients who were dialysis dependent and the 7 who died.

## Supplementary data 1: Participants

45 centres across the United Kingdom received local ethical and management approval for the study and had permission to register patients into the study (22 centres recruited at least 1 patient). Patients entered the study based on the following eligibility criteria:

### 1.1.1 Inclusion Criteria

- Newly diagnosed myeloma
- Acute renal failure (unresponsive to treatment with fluid and/or treatment of hypercalcaemia with bisphosphonate), attributable to myeloma
- Aged 18 years or over
- Written informed consent.

Renal biopsy was not required for trial entry but could be taken for reasons of diagnosis and management at the discretion of the local clinicians.

### 1.1.2 Exclusion Criteria

- Previous chemotherapy for myeloma (up to three days of steroid therapy was permissible; any more should be discussed with a trial investigator)
- Pregnancy
- Inadequate contraception
- Known HIV seropositivity (HIV testing was not a requirement for this study)
- Significant intrinsic renal disease unrelated to myeloma
- Platelet count <50x10^9^/l (risk of exacerbation of thrombocytopenia by plasma exchange)
- Standard contra-indications to study medication including:
  - Active or recent peptic ulcer
  - Known significant cardiac insufficiency
  - Abnormal liver function tests (bilirubin >1.5x upper limit of normal *and/or* ALT or AST >2.5x upper limit of normal)
  - Allergy to study medications.

## 1.2 Interventions

All patients should have received two four-day courses of dexamethasone. This was followed by VAD if randomised before the major protocol amendment or treatment with a chemotherapeutic regimen chosen by the local treating clinician, with appropriate supportive therapy, if randomised after the major protocol amendment (November 2005).

Days 1-4 inclusive Dexamethasone 40mg daily (orally) (given in 2 or more divided doses)

Days 9-12 inclusive Dexamethasone 40mg daily (orally) (given in 2 or more divided doses)

Days 17-20 inclusive Dexamethasone 40mg daily (orally) Vincristine 0.4mg daily by continuous IV infusion Adriamycin 9mg/m2 daily by continuous IV infusion

Days 38-41 inclusive Dexamethasone 40mg daily (orally) Vincristine 0.4mg daily by continuous IV infusion Adriamycin 9mg/m2 daily by continuous IV infusion

Days 59-62 inclusive Dexamethasone 40mg daily (orally) Vincristine 0.4mg daily by continuous IV infusion Adriamycin 9mg/m2 daily by continuous IV infusion

Days 80-83 inclusive Dexamethasone 40mg daily (orally) Vincristine 0.4mg daily by continuous IV infusion Adriamycin 9mg/m2 daily by continuous IV infusion.

In November 2005, when 46 patients had been entered into the trial, the protocol was amended so that from day 17 – 100 chemotherapy would be the choice of the local treating clinician rather than as the protocol previously stated should be VAD

Patients were randomised at entry to undergo seven plasma exchange treatments within the first two weeks, or to be treated with drugs alone.

Protocol stated patients will undergo seven plasma exchanges (by either cytocentrifugation or plasma filtration, according to local practice), each of 60ml/kg (maximum 4 litres), within two weeks of entry. At least four of the seven exchanges should take place in the first week.

Replacement fluid should be albumin unless administration of fresh frozen plasma is required to correct coagulation factor depletion. Physiological concentrations of potassium and calcium may be added to the albumin to maintain serum concentrations. Note: Calcium should not be coadministered with citrate anti-coagulation.

## 1.3 Randomisation

Randomisation was administered by the central trials office, the Clinical Trials and Research Unit (CTRU) at the University of Leeds, using an automated 24-hour telephone system. Treatment allocation was using a minimisation algorithm, and patients were randomised to receive either plasma exchange or no plasma exchange on a 1:1 basis.

Following the major protocol amendment, where chemotherapy given after the initial dexamethasone therapy would be the choice of the local treating clinician, the stratification factors used in the minimisation algorithm were changed in order to ensure balance in the appropriate factors between the treatment arms.

The stratification factors used in the minimisation algorithm before the amendment was implemented were:

- Centre
- The need or not for dialysis at the time of randomisation
- Age (<65, ≥65 years)

The stratification factors used in the minimisation algorithm after the amendment was implemented were:

- Chemotherapy type (VAD/VAD like, thalidomide containing, alkylating agent, other)
- Frequency of cycles (1-3 weekly, 4 weekly)
- The need or not for dialysis at randomisation
- Age (<65, ≥65 years)

### Supplementary Figure 1a: Study flow diagram

Patient completes baseline Quality of Life Questionnaire

Chemotherapy

&

Plasma exchange

Written consent

Newly diagnosed myeloma and acute renal failure

Randomised

Chemotherapy only

2 courses of Dexamethasone

(days 1-4, 9-12)

2 courses of Dexamethasone

(days 1-4, 9-12)

&

7 plasma exchange treatments

by cytocentrifugation or plasma filtration

(days 1-14; 4 in days 1-7)

VAD (patients randomised to protocol v1 only) or chemotherapy chosen by the local clinician (patients randomised to protocol v2 only)

up to Day 100

(first cycle beginning day 17) post randomisation

##

Treatment as per local protocol

(after 100 days)

### Supplementary Figure 1b: CONSORT flow diagram

Randomised (n=79)

Allocated to receive plasma exchange (n=39)

Received plasma exchange (n=36)

Did not receive plasma exchange (n=0)

No consent received (n=1)

Missing (n=2)

Allocated to receive no plasma exchange (n=40)

Received plasma exchange (n=0)

Did not receive plasma exchange (n=39)

Missing (n=1)

Lost to follow-up before 100 days (n=2)

(one reason missing, one patient withdrew)

Missing primary endpoint data (n=3)

Withdrew consent (n=1)

Before 100 days (n=1)

After 100 days (n=0)

Withdrew consent for collection of follow-up data (n=1)

Before 100 days (n=1)

After 100 days (n=0)

Withdrew consent for previously collected data to be included in the study (n=0)

Before 100 days (n=0)

After 100 days (n=0)

Lost to follow-up before 100 days (n=0)

Missing primary endpoint data (n=3)

Withdrew consent (n=0)

Before 100 days (n=0)

After 100 days (n=0)

Withdrew consent for collection of follow-up data (n=0)

Before 100 days (n=0)

After 100 days (n=0)

Withdrew consent for previously collected data to be included in the study (n=0)

Before 100 days (n=0)

After 100 days (n=0)

ITT Population (n=38)

(1 patient excluded due to no consent form being received)

Eligible for per-protocol population (n=29)

Consented for inclusion within quality of life population (n=36)

ITT Population (n=40)

Eligible for per-protocol population (n=40)

Consented for inclusion within quality of life population (n=39)

The intention to treat (ITT) population consists of 78 patients (38 Plasma exchange, 40 No Plasma exchange). This excludes one patient with no record of consent who was randomised to Plasma Exchange. The per protocol population consists of 69 patients (29 Plasma Exchange, 40 No Plasma Exchange) who were not considered protocol violators. Protocol violations include one patient with no record of consent, one patient who withdrew consent after 4 days and 8 patients in the plasma exchange group that did not receive 7 plasma exchanges.

Of the 37 patients randomised to plasma exchange with a record of consent that was not subsequently withdrawn, 8 did not receive the specified 7 cycles of plasma exchange. Instead they received 1, 3, 4, 4, 4, 5, 6 and 6 cycles before discontinuation. The respective reasons were:

- Adverse reaction to PE: Severe Vasovagal episode
- Patient too unwell: “Malena stools, gastric ulcer”, “Decreased clotting factors”
- Patient too unwell: “Chest infection”, “Died 25/05/08 MI”
- Patient too unwell: “Admitted to ICU with sepsis”
- Patient choice, abdominal discomfort and increased temperature
- Adverse reaction to PE: Thrombocytopenia
- Patient too unwell: Chest infection, GI bleeding
- Patient too unwell: No further details

The quality of life population (QOL) consists of 75 patients (36 Plasma Exchange, 39 No Plasma Exchange). This includes all patients that gave separate informed consent to be part of the QOL study.

# **SUPPLEMENTARY DATA 2**

## 2.1 Patient Demographics

The average age of patients overall was 67, patients in the plasma exchange group had a mean age of 67.2 while patients in the no plasma exchange group had a mean age of 66.8.

***Table 2.1.1: Age at randomisation***

| **Treatment group** | **N** | **Mean** | **Median** | **SD** | **SE** | **Min** | **Max** | **Missing** | **Total** |
| --- | --- | --- | --- | --- | --- | --- | --- | --- | --- |
| Plasma Exchange | 38 | 67.2 | 68.5 | 9.1 | 1.5 | 45 | 85 | 0 | 38 |
| No Plasma Exchange | 40 | 66.8 | 68.5 | 8.9 | 1.4 | 38 | 80 | 0 | 40 |
| All | 78 | 67.0 | 68.5 | 9.0 | 1.0 | 38 | 85 | 0 | 78 |

The proportion of males was 11% higher in the plasma exchange group compared with those in the no plasma exchange group.

***Table 2.1.2: Gender by treatment group and overall***

|  | **Gender** | |  |
| --- | --- | --- | --- |
| **Treatment group** | **Male** | **Female** | **Total** |
| Plasma Exchange | 24 (63.2%) | 14 (36.8%) | 38 |
| No Plasma Exchange | 21 (52.5%) | 19 (47.5%) | 40 |
| Total | 45 (57.7%) | 33 (42.3%) | 78 |

## 2.2 Renal Function

A total of 47(60%) patients required dialysis at randomisation, 22(58%) in the plasma exchange group and 25(63%) in the no plasma exchange group. Table 2.2.1 summarising patients requiring dialysis at baseline is based on complete data as this was collected as a stratification factor at randomisation for patients randomised to both version 1 and 2 of the protocol.

***Table 5.2.1: Patients requiring dialysis at baseline***

|  | **Patients Requiring Dialysis** | |  |
| --- | --- | --- | --- |
| **Treatment group** | **Yes** | **No** | **Total** |
| Plasma Exchange | 22 (57.9%) | 16 (42.1%) | 38 |
| No Plasma Exchange | 25 (62.5%) | 15 (37.5%) | 40 |
| Total | 47 (60.3%) | 31 (39.7%) | 78 |

Table 5.2.2 summarises weight as collected on the baseline CRF and indicates a reasonable balance between treatment groups. The mean weight was 76.5kg (77.9kg in the plasma exchange group and 74.8kg in the no plasma exchange group).

***Table 2.2.2: Weight at randomisation (kg) (Patients not on dialysis at baseline)***

| **Treatment group** | **N** | **Mean** | **Median** | **SD** | **SE** | **Min** | **Max** | **Missing** | **Total** |
| --- | --- | --- | --- | --- | --- | --- | --- | --- | --- |
| Plasma Exchange | 14 | 77.86 | 79.20 | 12.740 | 3.405 | 58.90 | 102.00 | 2 | 16 |
| No Plasma Exchange | 11 | 74.79 | 74.70 | 14.260 | 4.299 | 49.80 | 105.60 | 4 | 15 |
| All | 25 | 76.51 | 74.70 | 13.231 | 2.646 | 49.80 | 105.60 | 6 | 31 |

Only includes patients who were not receiving dialysis.

## 2.3 Hydration

Table 2.2.2 summarises patients undergoing re-hydration therapy as collected on the baseline CRF and indicates a slight imbalance between treatment groups. 16(42.1%) patients in the plasma exchange group and 22(55.0%) patients in the no plasma exchange treatment group underwent rehydration therapy before randomisation.

***Table 2.3.1: Re-hydration therapy before randomisation by treatment group and overall***

|  | **Rehydration therapy before randomisation** | | |  |
| --- | --- | --- | --- | --- |
| **Treatment group** | **Yes** | **No** | **Missing** | **Total** |
| Plasma Exchange | 16 (42.1%) | 19 (50.0%) | 3 (7.9%) | 38 |
| No Plasma Exchange | 22 (55.0%) | 16 (40.0%) | 2 (5.0%) | 40 |
| Total | 38 (48.7%) | 35 (44.9%) | 5 (6.4%) | 78 |

## 2.4 Baseline Local Laboratory Summaries

Local haematology, bone marrow aspirate and bio-chemistry laboratory results collected on the baseline CRF are summarised below.

The tables suggest that the baseline lab results are mostly well balanced between treatment group.

### 2.4.1 Haematology

***Table 2.4.1.1: Haemoglobin (g/dl)***

| **Treatment group** |  | **N** | **Mean** | **Median** | **SD** | **SE** | **Min** | **Max** | **Missing** | **Total** | **N(%) <11.5** | **N(%) >=11.5** |
| --- | --- | --- | --- | --- | --- | --- | --- | --- | --- | --- | --- | --- |
| Plasma Exchange | Male | 23 | 9.45 | 9.00 | 1.428 | 0.298 | 6.60 | 12.60 | 1 | 24 | 22 (95.7%) | 1 (4.3%) |
|  | Female | 14 | 9.09 | 9.05 | 1.335 | 0.357 | 6.40 | 11.50 | 0 | 14 | 12 (85.7%) | 2 (14.3%) |
| No Plasma Exchange | Male | 21 | 9.13 | 9.00 | 1.184 | 0.258 | 6.90 | 11.50 | 0 | 21 | 20 (95.2%) | 1 (4.8%) |
|  | Female | 19 | 9.14 | 8.90 | 1.180 | 0.271 | 7.30 | 11.40 | 0 | 19 | 16 (84.2%) | 3 (15.8%) |
| All | Male | 44 | 9.30 | 9.00 | 1.312 | 0.198 | 6.60 | 12.60 | 1 | 45 | 42 (95.5%) | 2 (4.5%) |
|  | Female | 33 | 9.12 | 9.00 | 1.228 | 0.214 | 6.40 | 11.50 | 0 | 33 | 28 (84.8%) | 5 (15.2%) |

***Table 2.4.1.2: WBC (x 10^9^/l)***

| **Treatment group** | **N** | **Mean** | **Median** | **SD** | **SE** | **Min** | **Max** | **Missing** | **Total** |
| --- | --- | --- | --- | --- | --- | --- | --- | --- | --- |
| Plasma Exchange | 37 | 8.64 | 7.40 | 4.510 | 0.741 | 2.50 | 21.10 | 1 | 38 |
| No Plasma Exchange | 40 | 7.96 | 7.05 | 4.133 | 0.653 | 2.80 | 22.30 | 0 | 40 |
| All | 77 | 8.29 | 7.10 | 4.303 | 0.490 | 2.50 | 22.30 | 1 | 78 |

***Table 2.4.1.3: Neutrophils (x 10^9^/l)***

| **Treatment group** | **N** | **Mean** | **Median** | **SD** | **SE** | **Min** | **Max** | **Missing** | **Total** | **N(%) <1.5** | **N(%) >=1.5** |
| --- | --- | --- | --- | --- | --- | --- | --- | --- | --- | --- | --- |
| Plasma Exchange | 37 | 6.06 | 4.90 | 3.364 | 0.553 | 1.50 | 12.90 | 1 | 38 | 0 (0.0%) | 37 (100.0%) |
| No Plasma Exchange | 40 | 5.64 | 4.70 | 3.881 | 0.614 | 1.50 | 21.30 | 0 | 40 | 0 (0.0%) | 40 (100.0%) |
| All | 77 | 5.84 | 4.90 | 3.624 | 0.413 | 1.50 | 21.30 | 1 | 78 | 0 (0.0%) | 77 (100.0%) |

***Table 2.4.1.4: Platelets (x 10^9^/l)***

| **Treatment group** | **N** | **Mean** | **Median** | **SD** | **SE** | **Min** | **Max** | **Missing** | **Total** | **N(%) <140** | **N(%) >=140** |
| --- | --- | --- | --- | --- | --- | --- | --- | --- | --- | --- | --- |
| Plasma Exchange | 37 | 201.03 | 199.00 | 72.700 | 11.952 | 79.00 | 408.00 | 1 | 38 | 8 (21.6%) | 29 (78.4%) |
| No Plasma Exchange | 40 | 210.08 | 198.50 | 67.773 | 10.716 | 97.00 | 376.00 | 0 | 40 | 7 (17.5%) | 33 (82.5%) |
| All | 77 | 205.73 | 199.00 | 69.866 | 7.962 | 79.00 | 408.00 | 1 | 78 | 15 (19.5%) | 62 (80.5%) |

### 2.4.2 Bone Marrow Aspirate

***Table 2.4.2.1: Bone marrow plasma cell percentage (%)***

| **Treatment group** | **N** | **Mean** | **Median** | **SD** | **SE** | **Min** | **Max** | **Missing** | **Total** | **N(%) <20** | **N(%) >=20** |
| --- | --- | --- | --- | --- | --- | --- | --- | --- | --- | --- | --- |
| Plasma Exchange | 34 | 41.06 | 38.00 | 25.472 | 4.368 | 5.00 | 95.00 | 4 | 38 | 8 (23.5%) | 26 (76.5%) |
| No Plasma Exchange | 38 | 47.81 | 50.00 | 26.172 | 4.246 | 3.00 | 92.00 | 2 | 40 | 8 (21.1%) | 30 (78.9%) |
| All | 72 | 44.62 | 48.50 | 25.885 | 3.051 | 3.00 | 95.00 | 6 | 78 | 16 (22.2%) | 56 (77.8%) |

### 2.4.3 Biochemistry

Serum creatinine, as summarised in table 5.4.3.1, was only collected for the 31 patients who were dialysis independent at baseline. Glomerular Filtration Rate (GFR) was calculated via Cockcroft-Gault and MDRD formulas, which are both based on serum creatinine; therefore tables 5.4.3.4 and 5.4.3.5 are also based on the 31 dialysis independent patients only.

Within the 31 dialysis independent patients at baseline, patients in the plasma exchange group had an average GFR (according to the Cockcroft-Gault formula) of 12.2 while patients in the no plasma exchange group had an average GFR of 10.8, although this is based on only 25 patients with non-missing data. This average was also higher in the plasma exchange group when calculated according to the MDRD formula.

***Table 2.4.3.1: Serum creatinine (µmol/l)***

| **Treatment group** | **N** | **Mean** | **Median** | **SD** | **SE** | **Min** | **Max** | **Total** |
| --- | --- | --- | --- | --- | --- | --- | --- | --- |
| Plasma Exchange | 16 | 591.63 | 571.00 | 86.504 | 21.626 | 503.00 | 770.00 | 16 |
| No Plasma Exchange | 15 | 618.47 | 599.00 | 97.993 | 25.302 | 495.00 | 841.00 | 15 |
| All | 31 | 604.61 | 578.00 | 91.699 | 16.470 | 495.00 | 841.00 | 31 |

Only includes patients who were not receiving dialysis.

***Table 2.4.3.2: Serum calcium (corrected) (mmol/l)***

| **Treatment group** | **N** | **Mean** | **Median** | **SD** | **SE** | **Min** | **Max** | **Missing** | **Total** | **N(%) <2.6** | **N(%) >=2.6** |
| --- | --- | --- | --- | --- | --- | --- | --- | --- | --- | --- | --- |
| Plasma Exchange | 36 | 2.44 | 2.39 | 0.308 | 0.051 | 1.96 | 3.73 | 2 | 38 | 28 (77.8%) | 8 (22.2%) |
| No Plasma Exchange | 40 | 2.52 | 2.45 | 0.391 | 0.062 | 1.97 | 4.08 | 0 | 40 | 28 (70.0%) | 12 (30.0%) |
| All | 76 | 2.48 | 2.43 | 0.354 | 0.041 | 1.96 | 4.08 | 2 | 78 | 56 (73.7%) | 20 (26.3%) |

***Table 2.4.3.3: Serum albumin (g/l)***

| **Treatment group** | **N** | **Mean** | **Median** | **SD** | **SE** | **Min** | **Max** | **Missing** | **Total** | **N(%) <37** | **N(%) >=37** |
| --- | --- | --- | --- | --- | --- | --- | --- | --- | --- | --- | --- |
| Plasma Exchange | 37 | 37.76 | 39.00 | 7.025 | 1.155 | 22.00 | 53.00 | 1 | 38 | 17 (45.9%) | 20 (54.1%) |
| No Plasma Exchange | 40 | 36.30 | 35.50 | 6.313 | 0.998 | 25.00 | 50.00 | 0 | 40 | 24 (60.0%) | 16 (40.0%) |
| All | 77 | 37.00 | 36.00 | 6.661 | 0.759 | 22.00 | 53.00 | 1 | 78 | 41 (53.2%) | 36 (46.8%) |

***Table 2.4.3.4: Glomerular filtration rate (GFR) according to the Cockcroft-Gault formula***

| **Treatment group** | **N** | **Mean** | **Median** | **SD** | **SE** | **Min** | **Max** | **Missing** | **Total** |
| --- | --- | --- | --- | --- | --- | --- | --- | --- | --- |
| Plasma Exchange | 14 | 12.24 | 11.69 | 3.325 | 0.889 | 8.26 | 19.34 | 2 | 16 |
| No Plasma Exchange | 11 | 10.78 | 11.36 | 3.074 | 0.927 | 6.74 | 18.31 | 4 | 15 |
| All | 25 | 11.60 | 11.45 | 3.237 | 0.647 | 6.74 | 19.34 | 6 | 31 |

Only includes patients who were not receiving dialysis.

***Table 2.4.3.5: Glomerular filtration rate (GFR) according to the MDRD formula***

| **Treatment group** | **N** | **Mean** | **Median** | **SD** | **SE** | **Min** | **Max** | **Missing** | **Total** |
| --- | --- | --- | --- | --- | --- | --- | --- | --- | --- |
| Plasma Exchange | 10 | 8.94 | 9.59 | 1.671 | 0.529 | 6.54 | 10.84 | 6 | 16 |
| No Plasma Exchange | 8 | 8.32 | 8.64 | 1.654 | 0.585 | 5.78 | 10.11 | 7 | 15 |
| All | 18 | 8.67 | 9.12 | 1.645 | 0.388 | 5.78 | 10.84 | 13 | 31 |

Only includes patients who were not receiving dialysis.

Glomerular filtration rate was calculated according to the Cockcroft-Gault and MDRD formulas as shown in section 11.

## 2.5 Baseline Beta-2 microglobulin and international staging

The international staging system categorises myeloma patients into disease stages based on β2-microglobulin and serum albumin results according to the following criteria:

- Stage I: β2-microglobulin (β2M) < 3.5 mg/L, serum albumin >= 3.5 g/dL
- Stage II: β2M < 3.5 mg/L and albumin < 3.5 g/dL; or β2M 3.5 mg/L - 5.5 mg/L irrespective of the serum albumin
- Stage III: β2M >= 5.5 mg/L

Table 2.5.1 summarises β2-microglobulin at baseline, table 5.4.3.3 in the previous section shows a summary of serum albumin. 69(88.5%) patients at baseline fell into the category of stage III, the most advanced stage and only 1(1.3%) patient fell into the category of stage II. The remaining 8(10.3%) patients had missing ISS at baseline, mainly due to missing β2-microglobulin results.

***Table 2.5.1: Beta-2 microglobulin (mg/L)***

| **Treatment group** | **N** | **Mean** | **Median** | **SD** | **SE** | **Min** | **Max** | **Missing** | **Total** |
| --- | --- | --- | --- | --- | --- | --- | --- | --- | --- |
| Plasma Exchange | 33 | 28.75 | 22.10 | 18.990 | 3.306 | 6.90 | 88.90 | 5 | 38 |
| No Plasma Exchange | 37 | 31.16 | 24.40 | 18.185 | 2.990 | 2.80 | 82.10 | 3 | 40 |
| All | 70 | 30.02 | 24.05 | 18.473 | 2.208 | 2.80 | 88.90 | 8 | 78 |

***Table 2.5.3: International staging system (ISS)***

| **Treatment group** | **I** | **II** | **III** | **Missing** | **Total** |
| --- | --- | --- | --- | --- | --- |
| Plasma Exchange | 0 (0.0%) | 0 (0.0%) | 33 (86.8%) | 5 (13.2%) | 38 |
| No Plasma Exchange | 0 (0.0%) | 1 (2.5%) | 36 (90.0%) | 3 (7.5%) | 40 |
| Total | 0 (0.0%) | 1 (1.3%) | 69 (88.5%) | 8 (10.3%) | 78 |

# **Supplementary data 3:**

# **3.1 Primary Endpoint: Proportion Alive and Dialysis Independent at 100 Days**

The primary endpoint is patients alive and dialysis-independent at 100 days. Out of the 78 patients randomised, 6 (7.7%) patients had missing outcome (3 in each treatment group). 11 (28.9%) of patients in the Plasma Exchange group were alive and dialysis independent at 100 days compared with 11 (27.5%) in the No Plasma Exchange group.

***Table 3.1.1: Proportion alive and dialysis–independent 100 days after randomisation***

| **Treatment group** | **Alive and Dialysis-Independent** | **Dead** | **Dialysis-dependent** | **Missing** | **N** |
| --- | --- | --- | --- | --- | --- |
| Plasma Exchange | 11 (28.9%) | 7 (18.4%) | 17 (44.7%) | 3 (7.9%) | 38 |
| No Plasma Exchange | 11 (27.5%) | 5 (12.5%) | 21 (52.5%) | 3 (7.5%) | 40 |
| Total | 22 (28.2%) | 12 (15.4%) | 38 (48.7%) | 6 (7.7%) | 78 |

Table 3.1 summarises the number of patients alive and dialysis independent excluding the 6 (7.7%) patients with missing outcome. 31.4% (95% CI 16%-46.8%) of patients in the Plasma Exchange group were alive and dialysis independent at day 100 compared with 29.7% (95% CI 15.0%-44.4%) in the No Plasma Exchange group. There is no evidence of a difference between the treatment groups (p=0.876, and the confidence intervals have a large overlap).

## Proportion Alive and Dialysis Independent at 6 months

Out of the 78 patients randomised, 6 (7.7%) patients had a missing outcome of alive and dialysis independence at 6 months (3 in each treatment group and consistent with the primary analysis). 14/38 (36.8%) patients in the Plasma Exchange group were alive and dialysis independent at 6 months compared with 9/40 (22.5%) in the No Plasma Exchange group.

***Table 3.1.2: Proportion alive and dialysis–independent 6 months after randomisation***

| **Treatment group** | **Alive and Dialysis-Independent** | **Dead** | **Dialysis-dependent** | **Missing** | **N** |
| --- | --- | --- | --- | --- | --- |
| Plasma Exchange | 14 (36.8%) | 9 (23.7%) | 12 (31.6%) | 3 (7.9%) | 38 |
| No Plasma Exchange | 9 (22.5%) | 10 (25.0%) | 18 (45.0%) | 3 (7.5%) | 40 |
| Total | 23 (29.5%) | 19 (24.4%) | 30 (38.5%) | 6 (7.7%) | 78 |

Table 3.2 summarises the number of patients alive and dialysis independent excluding the 6 (7.7%) patients with missing outcome so that a 95% confidence interval can be derived. 40% (95% CI 23.7% - 56.2%) of patients in the Plasma Exchange group were alive and dialysis independent compared with 24.3% (95% CI 10.4% - 38.1%) in the No Plasma Exchange group. This difference is non-significant with a p-value of 0.154.

## Proportion Alive and Dialysis Independent at 12 months

Out of the 78 patients randomised, 8 (10.3%) patients had missing outcome of alive and dialysis independence at 12 months (4 in each treatment group). 9/38 (23.7%) patients in the Plasma Exchange group were alive and dialysis independent at 12 months compared with 7/40 (17.5%) in the No Plasma Exchange group.

***Table 3.1.3: Proportion alive and dialysis–independent 12 months after randomisation***

| **Treatment group** | **Alive and Dialysis-Independent** | **Dead** | **Dialysis-dependent** | **Missing** | **N** |
| --- | --- | --- | --- | --- | --- |
| Plasma Exchange | 9 (23.7%) | 15 (39.5%) | 10 (26.3%) | 4 (10.5%) | 38 |
| No Plasma Exchange | 7 (17.5%) | 20 (50.0%) | 9 (22.5%) | 4 (10.0%) | 40 |
| Total | 16 (20.5%) | 35 (44.9%) | 19 (24.4%) | 8 (10.3%) | 78 |

Table 3.3 summarises the number of patients alive and dialysis independent excluding the 8 (10.3%) patients with missing outcome so that a 95% confidence interval can be derived. 26.5% (95% CI 11.6% - 41.2%) of patients in the Plasma Exchange group were alive and dialysis independent compared with 19.4% (95% CI 6.5% - 32.3%) in the No Plasma Exchange group. This difference is non-significant with a p-value of 0.484.

## 3.2 Summary of malignant serum free light chain levels up to day 15

77 out of 78 patients had at least 1 free light chain result from any time point; therefore 1 patient’s malignant light chain type could not be derived as shown in table 3.2.1. Malignant light chain types are balanced overall and across treatment groups.

Tables 3.2.2 – 3.2.5 show raw summaries of malignant free light chain results (kappa or lambda) at baseline, day 5, day 10 and day 15-17 spanning the plasma exchange treatment period for those randomised to plasma exchange and no plasma exchange; statistics are shown for both FLC and log-FLC values (note, means are based on log-FLC and then back transformed). Tables 12.2.6 – 12.2.8 show absolute changes in log-malignant free light chain results from baseline. Tables 12.2.9 - 12.2.11 show percentage change in log-malignant free light chain results from baseline. Totals are based on the number of expected malignant free light chain results at that time point excluding patients that have died or withdrawn.

69/78(88.5%) patients have a baseline malignant free light chain result. Table 12.2.2 suggests, there is no evidence of a clear difference between the treatment groups at baseline.

***Table 12.1.1: Summary malignant free light chains (kappa or lambda) by treatment group***

|  | **Malignant chain** | | |  |
| --- | --- | --- | --- | --- |
| **Treatment group** | **Kappa** | **Lambda** | **Missing** | **Total** |
| Plasma Exchange | 19 (50.0%) | 18 (47.4%) | 1 (2.6%) | 38 |
| No Plasma Exchange | 20 (50.0%) | 20 (50.0%) | 0 (0.0%) | 40 |
| Total | 39 (50.0%) | 38 (48.7%) | 1 (1.3%) | 78 |

***Table 12.2.2: Summary of malignant free light chain results (mg/l) by treatment group at baseline***

|  | | **FLC** | | | | **log-FLC** | | | |  | |
| --- | --- | --- | --- | --- | --- | --- | --- | --- | --- | --- | --- |
| **Treatment group** | **N** | **Mean*** | **Median** | **Min** | **Max** | **Mean** | **Median** | **SD** | **SE** | **Missing** | **Total** |
| Plasma Exchange | 33 | 5956 | 5940 | 800 | 44112 | 8.69 | 8.69 | 1.165 | 0.203 | 5 | 38 |
| No Plasma Exchange | 36 | 6304 | 6632 | 1320 | 57695 | 8.75 | 8.80 | 0.850 | 0.142 | 4 | 40 |
| Total | 69 | 6136 | 6319 | 800 | 57695 | 8.72 | 8.75 | 1.005 | 0.121 | 9 | 78 |

*Mean based on log-FLC and then back transformed

***Table 12.2.3: Summary of malignant free light chain (mg/l) results by treatment group on day 5***

|  | | **FLC** | | | | **log-FLC** | | | |  | |
| --- | --- | --- | --- | --- | --- | --- | --- | --- | --- | --- | --- |
| **Treatment group** | **N** | **Mean*** | **Median** | **Min** | **Max** | **Mean** | **Median** | **SD** | **SE** | **Missing** | **Total** |
| Plasma Exchange | 32 | 3196 | 4090 | 191 | 32767 | 8.07 | 8.32 | 1.323 | 0.234 | 5 | 37 |
| No Plasma Exchange | 34 | 3600 | 4018 | 405 | 17668 | 8.19 | 8.30 | 1.004 | 0.172 | 5 | 39 |
| Total | 66 | 3398 | 4090 | 191 | 32767 | 8.13 | 8.32 | 1.162 | 0.143 | 10 | 76 |

*Mean based on log-FLC and then back transformed

***Table 12.2.4: Summary of malignant free light chain results (mg/l) by treatment group on day 10***

|  | | **FLC** | | | | **log-FLC** | | | |  | |
| --- | --- | --- | --- | --- | --- | --- | --- | --- | --- | --- | --- |
| **Treatment group** | **N** | **Mean*** | **Median** | **Min** | **Max** | **Mean** | **Median** | **SD** | **SE** | **Missing** | **Total** |
| Plasma Exchange | 33 | 2540 | 1829 | 267 | 19187 | 7.84 | 7.51 | 1.306 | 0.227 | 3 | 36 |
| No Plasma Exchange | 34 | 2751 | 3035 | 228 | 22077 | 7.92 | 8.01 | 1.215 | 0.208 | 4 | 38 |
| Total | 67 | 2645 | 2414 | 228 | 22077 | 7.88 | 7.79 | 1.252 | 0.153 | 7 | 74 |

*Mean based on log-FLC and then back transformed

***Table 12.2.5: Summary of malignant free light chain results (mg/l) by treatment group on day 15-17***

|  | | **FLC** | | | | **log-FLC** | | | |  | |
| --- | --- | --- | --- | --- | --- | --- | --- | --- | --- | --- | --- |
| **Treatment group** | **N** | **Mean*** | **Median** | **Min** | **Max** | **Mean** | **Median** | **SD** | **SE** | **Missing** | **Total** |
| Plasma Exchange | 26 | 2783 | 2945 | 195 | 32891 | 7.93 | 7.97 | 1.580 | 0.310 | 10 | 36 |
| No Plasma Exchange | 33 | 2198 | 1976 | 234 | 20459 | 7.70 | 7.59 | 1.204 | 0.210 | 5 | 38 |
| Total | 59 | 2439 | 2441 | 195 | 32891 | 7.80 | 7.80 | 1.375 | 0.179 | 15 | 74 |

*Mean based on log-FLC and then back transformed

Tables 12.2.6 – 12.2.8 show a mean decrease in log-FLC of 0.76 between baseline and day 5 (0.82 in the plasma exchange group and 0.70 in the no plasma exchange group). This decrease increases at day 10 and day 15-17. When transforming back on to the original FLC scale, these results imply that a patient with a baseline malignant FLC result of 10,000mg/l (for example) will on average decrease to 4,677mg/l at day 5 (4404mg/l in the plasma exchange group and 4966mg/l in the no plasma exchange group); 3,829mg/l at day 10 (3642mg/l in the plasma exchange group and 4066mg/l in the no plasma exchange group); and 3,535mg/l at day 15/17 (3396mg/l in the plasma exchange group and 3606mg/l in the no plasma exchange group).

***Table 12.2.6: Absolute change in log-malignant free light chain results (mg/l) from baseline on day 5***

| **Treatment group** | **N** | **Mean** | **Median** | **SD** | **SE** | **Min** | **Max** | **Missing** | **Total** |
| --- | --- | --- | --- | --- | --- | --- | --- | --- | --- |
| Plasma Exchange | 29 | 0.82 | 0.68 | 0.851 | 0.158 | -0.41 | 3.72 | 8 | 37 |
| No Plasma Exchange | 31 | 0.70 | 0.65 | 0.813 | 0.146 | -0.81 | 3.58 | 8 | 39 |
| Total | 60 | 0.76 | 0.65 | 0.826 | 0.107 | -0.81 | 3.72 | 16 | 76 |

***Table 12.2.7: Absolute change in log-malignant free light chain results (mg/l) from baseline by treatment group on day 10***

| **Treatment group** | **N** | **Mean** | **Median** | **SD** | **SE** | **Min** | **Max** | **Missing** | **Total** |
| --- | --- | --- | --- | --- | --- | --- | --- | --- | --- |
| Plasma Exchange | 29 | 1.01 | 0.92 | 1.033 | 0.192 | -0.61 | 3.50 | 7 | 36 |
| No Plasma Exchange | 31 | 0.90 | 0.76 | 0.982 | 0.176 | -0.58 | 3.86 | 7 | 38 |
| Total | 60 | 0.96 | 0.80 | 1.000 | 0.129 | -0.61 | 3.86 | 14 | 74 |

***Table 12.2.8: Absolute change in log-malignant free light chain results (mg/l) from baseline by treatment group on day 15-17***

| **Treatment group** | **N** | **Mean** | **Median** | **SD** | **SE** | **Min** | **Max** | **Missing** | **Total** |
| --- | --- | --- | --- | --- | --- | --- | --- | --- | --- |
| Plasma Exchange | 23 | 1.08 | 1.11 | 0.973 | 0.203 | -1.03 | 3.81 | 13 | 36 |
| No Plasma Exchange | 29 | 1.02 | 0.73 | 1.063 | 0.197 | -0.52 | 4.30 | 9 | 38 |
| Total | 52 | 1.04 | 0.97 | 1.015 | 0.141 | -1.03 | 4.30 | 22 | 74 |

Tables 12.2.9 – 12.2.11 show percentage change in malignant FLC from baseline, and suggest consistent results with absolute changes from baseline. The mean decrease in log-FLC between day 5 and baseline was 8.43 (9.15 in the plasma exchange group and 7.74 in the no plasma exchange group).

***Table 12.2.9: Percentage change in log-malignant free light chain results (mg/l) from baseline on day 5***

| **Treatment group** | **N** | **Mean** | **Median** | **SD** | **SE** | **Min** | **Max** | **Missing** | **Total** |
| --- | --- | --- | --- | --- | --- | --- | --- | --- | --- |
| Plasma Exchange | 29 | 9.15 | 6.97 | 9.619 | 1.786 | -5.21 | 41.45 | 8 | 37 |
| No Plasma Exchange | 31 | 7.74 | 7.64 | 8.516 | 1.530 | -9.71 | 32.69 | 8 | 39 |
| Total | 60 | 8.43 | 7.42 | 9.016 | 1.164 | -9.71 | 41.45 | 16 | 76 |

***Table 12.2.10: Percentage change in log-malignant free light chain results (mg/l) from baseline by treatment group on day 10***

| **Treatment group** | **N** | **Mean** | **Median** | **SD** | **SE** | **Min** | **Max** | **Missing** | **Total** |
| --- | --- | --- | --- | --- | --- | --- | --- | --- | --- |
| Plasma Exchange | 29 | 11.33 | 11.03 | 11.274 | 2.094 | -7.75 | 38.51 | 7 | 36 |
| No Plasma Exchange | 31 | 10.23 | 7.74 | 10.686 | 1.919 | -7.15 | 35.18 | 7 | 38 |
| Total | 60 | 10.76 | 10.07 | 10.895 | 1.406 | -7.75 | 38.51 | 14 | 74 |

***Table 12.2.11: Percentage change in log-malignant free light chain results (mg/l) from baseline by treatment group on day 15-17***

| **Treatment group** | **N** | **Mean** | **Median** | **SD** | **SE** | **Min** | **Max** | **Missing** | **Total** |
| --- | --- | --- | --- | --- | --- | --- | --- | --- | --- |
| Plasma Exchange | 23 | 12.38 | 12.65 | 11.419 | 2.381 | -12.95 | 41.98 | 13 | 36 |
| No Plasma Exchange | 29 | 11.46 | 8.22 | 11.471 | 2.130 | -6.58 | 39.25 | 9 | 38 |
| Total | 52 | 11.87 | 10.38 | 11.345 | 1.573 | -12.95 | 41.98 | 22 | 74 |
